# Prefabricated and Self-Setting Cement Laminates

**DOI:** 10.3390/ma12050834

**Published:** 2019-03-12

**Authors:** Theresa Brückner, Andreas Fuchs, Laura Wistlich, Andreas Hoess, Berthold Nies, Uwe Gbureck

**Affiliations:** 1 Department for Functional Materials in Medicine and Dentistry, University Hospital Würzburg, Pleicherwall 2, 97070 Würzburg, Germany; theresa.brueckner@fmz.uni-wuerzburg.de (T.B.); laura.wistlich@fmz.uni-wuerzburg.de (L.W.); 2 Department of Oral & Maxillofacial Plastic Surgery, University Hospital Würzburg, Pleicherwall 2, 97070 Würzburg, Germany; Fuchs_A2@ukw.de; 3 INNOTERE GmbH, Meissner Strasse 191, 01445 Radebeul, Germany; andreas.hoess@innotere.de (A.H.); berthold.nies@innotere.de (B.N.)

**Keywords:** calcium phosphate cement, prefabricated paste, electrospinning, laminate

## Abstract

Polycaprolactone (PCL) fiber mats with defined pore architecture were shown to provide sufficient support for a premixed calcium phosphate cement (CPC) paste to serve as a flat and flexible composite material for the potential application in 2-dimensional, curved cranial defects. Fiber mats were fabricated by either melt electrospinning writing (MEW) or solution electrospinning (SES) with a patterned collector. While MEW processed fiber mats led to a deterioration of the cement bending strength by approximately 50%, due to a low fiber volume content in conjunction with a weak fiber-matrix interface, fiber mats obtained by solution electrospinning resulted in a mechanical reinforcement of the cement matrix in terms of both bending strength and absorbed fracture energy. This was attributed to a higher fiber volume content and a large contact area between nanosized fibers and cement matrix. Hydrophilization of the PCL scaffolds prior to lamination further improved composite strength and preserved the comparably higher fracture energy of 1.5 to 2.0 mJ/mm^2^. The laminate composite approach from this study was successful in demonstrating the limitations and design options of such novel composite materials. However, fiber-cement compatibility remains an issue to be addressed, since a high degree of hydrophilicity does not necessarily provoke a stronger interface.

## 1. Introduction

Mineral bone cements mainly consisting of calcium phosphate phases are clinically well-established materials for bone replacement. Such cements are usually applied as a paste by either injection or modelling with a spatula [1]. As long as CPC still undergo setting from a pasty into the solid state, they need a surrounding supportive structure to maintain pre-modelled implant geometry. Thus, they are preferably used in cavity-like defects, which is additionally emphasized by their classification as “bone void fillers” [2]. In contrast, flat defects (e.g., in cranioplasty) are mostly treated with intraoperatively moulded polymethylmethacrylate (PMMA) cement implants [3,4], custom made titanium, polyethylene (PE) or polyetheretherketone (PEEK) implants, titanium meshes as a support for cementitious pastes or preformed solid calcium phosphate implants [5]. The former are known for damaging native tissue due to their heat development and monomer release, while metal implants suffer from their high thermal conductivity [6] and low malleability [7]. On the other hand, prefabricated implants are highly accurate in restoring the anatomical contour, but at the same time they are cost- and time intensive to produce. All implants share a certain risk for post-treatment infection, which is found to be larger for PMMA and titanium implants (~8–11%) than for hydroxyapatite materials (~2%) [5].

In this context, the current study is a proof of concept for the fabrication of self-setting laminates derived from a premixed calcium phosphate cement paste and polymeric fiber mats produced by electrospinning. Such laminates combine the unique properties of flexible polymer structures and a mineral bone cement preferably addressing the treatment of planar or curved cranial defects. In a recent approach, comparable laminates were fabricated with aqueous cement pastes, which left only limited time during clinical application when preparing the composite intraoperatively and adapting it to a bone defect [8,9]. Here, we used an oil-based premixed cement paste to overcome this limitation [10,11]. The paste was stabilized by layers of degradable polymer mats so that the cement paste has a 2-dimensionally interconnected support structure, which keeps it in place and retains its flexibility and adaptability to flat or curvy defects. Due to the unique paste properties the material maintains its moldability when stored under dry conditions. In contact with an aqueous environment, e.g., in the bone defect, the oil phase is then exchanged by water diffusion initiating the cement setting reaction. The reinforcing fiber mats were fabricated from PCL by either melt electrospinning writing (MEW) or solution electrospinning (SES) [12]. The influence of processing regime, areal density and post-treatment under alkaline conditions on the handling, mechanical properties and interphase between cementitious matrix and polymer fibers was investigated. Ideally, the fiber mats can initially reinforce the hardened cement and—as being biodegradable—leave interconnected cavities for an improved resorption and ingrowth of newly formed tissue.

## 2. Materials and Methods 

### 2.1. Melt Electrospinning Writing (MEW)

Melt electrospinning written PCL-scaffolds (50 × 50 mm^2^) from Purac PCL (PURASORB PC12, Corbion, Amsterdam, Netherlands) with box-shaped structure (fiber space: 200, 500 or 1000 µm; fiber diameter: 8 µm; layer number: 30) were produced by a custom made MEW device as described elsewhere [13].

### 2.2. Perforated Solution Electrospinning (SES)

306 mg Purac PCL (PURASORB PC12, Corbion, Amsterdam, Netherlands) was dissolved in 800 µL dichloromethane. 200 µL ethanol was added to this solution and stirred for at least 5 min. The polymer solution was transferred into a 1 mL syringe which was placed on a syringe pump (11Plus, Harvard Apparatus, Holliston, MA, USA). Solution electrospinning was performed with a blunt 27 G stainless steel cannula and a pumping speed of 0.5 mL/h. A high voltage of 12 kV (PS 2403D power supply, Voltcraft, Conrad, Hirschau, Germany) was applied at the spinneret tip. The charged threads of the polymer solution were then deposited on a rotating, grounded collector (Ø 80 mm) perforated with round holes in alternating rows with a hole diameter of 1 mm, a hole distance of 2 mm and a hole depth of 1 mm. After the spinning process, the scaffold was relieved from the collector with water, dried on air, and cut into 50 × 50 mm^2^ square pieces.

### 2.3. Surface Treatment & Contact Angle Measurement

The SES scaffolds were each incubated in 1.0 or 2.0 M NaOH for 10, 30 or 60 min under shaking (50 rpm) at RT. The NaOH-to-area ratio was 1 mL/cm^2^. Afterwards, the scaffolds were washed three times in the same amount of water at 50 rpm for 5 min and dried overnight at RT. Contact angles of the scaffolds were measured with the system OCA20 (Dataphysics, Filderstadt, Germany) using a dispense dosage of 3 µL and a dosing rate of 1.0 µL/s. Each measurement was performed thrice on the smooth surface (drop above pore and between pores).

### 2.4. Laminate Fabrication

**Figure 1:**
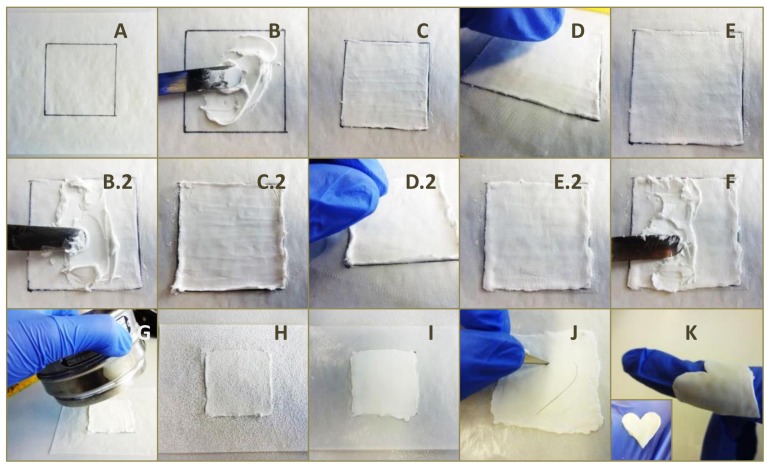
Manufacturing regime of prefabricated laminates using three layers of MEW PCL scaffolds with a fiber distance of 500 µm. 1.5 g of the cement paste is smeared evenly on a 50 × 50 mm^2^ weight paper (**A**–**C**) with a PCL scaffold pressed on top (**D**,**E**) followed by a second layer of cement paste and PCL scaffold (**B.2**–**E.2**). This procedure is repeated three times with a last cement paste layer on top (**F**). Lastly, α-TCP is sieved through a 300 µm sieve on the laminate (**G**,**H**) and spread to evenly cover all oil residues on top and bottom (**I**). The fabricated laminate can be cut into arbitrary, flexible form (**J**,**K**).

A premixed cement paste (INNOTERE Paste-CPC) was kindly provided by INNOTERE GmbH (Radebeul, Germany) consisting of the Biocement D raw powder mixture, a water-immiscible synthetic triglyceride (Mygliol 812) and a mixture of two surfactants (castor oil ethoxylate 35 and hexadecyl phosphate) [10]. The cement paste is known to convert into low crystalline Hydroxyapatite after immersion into an aqueous electrolyte. The laminate preparation regime is depicted in Figure 1. Briefly, 1.5 g of the cement paste was smeared with a spatula evenly on a weight paper (50 × 50 mm^2^). A PCL scaffold was pressed onto the cement layer and a second layer was smeared evenly on top. This procedure was repeated until three to seven layers of PCL scaffolds were laminated, with a last cement paste layer on top. In the case of MEW scaffolds, excess cement of each layer was removed by exfoliation with a sheet of aluminum foil. Each cement layer had a weight of about 1.5 g (MEW scaffolds) or 2.0 g (perforated SES scaffolds), respectively. Finally, α-TCP powder was sieved through a 300 µm sieve on top of the laminate and spread, so that all oil residues on top were covered evenly. The laminate was separated from the weight paper and the backside was also covered with α-TCP powder. Excess of raw powder was removed using compressed air.

### 2.5. Testing Methods

The prefabricated laminates were cut into bars with a size of 4 × 45 mm^2^ using two parallel cutter blades (n = 10), stored for 24 h at 37 °C and >90% humidity and finally cured in water for another 24 h at 37 °C.

Force-displacement curves were recorded in a 4-point-bending test setup according to ISO 6872 on a universal testing machine with a crosshead speed of 1 mm/min and a 2.5 kN load cell and used to calculate bending strength *σ_b_* (MPa) and bending modulus *E* (MPa) as follows (Equations (1) and (2)):

(1) $$\sigma_{b}=\frac{3\times l_{A}\times F_{max}}{b\times a^{2}}$$

(2) $$E=\frac{3\times l_{A}\times l_{B}^{2}\times \left(F_{H}-F_{L}\right)}{4\times X\times b\times a^{3}}$$

where *l_A_* = 10 mm is the span length, *l_B_* = 20 mm is the length of the reference bar, *F_max_* (N) is the maximum occurring force, *F_H_* (N) is the end and *F_L_* (N) the starting force of the bending modulus calculation, *X* (mm) is the bar bending, *b* (mm) is the bar width and *a* (mm) is the bar height. The areas under the stress-displacement-curves were calculated up to *σ_b_* as a measure for fracture energy.

Scanning electron microscopy (SEM) of the fiber mats and the cement laminates was performed with a crossbeam scanning electron microscope (SEM, CB 340, Carl Zeiss Microscopy GmbH, Oberkochen, Germany) with an acceleration voltage of 2 kV. Before analysis, the samples were sputtered with a 4 nm platinum layer.

### 2.6. Statistics

Significant differences (p < 0.001) were investigated performing a one-way analysis of variance (1-way ANOVA) with an all pairwise multiple comparison procedure (Tukey Test) using the software SigmaPlot (v12, Systat Software, Erkrath, Germany).

## 3. Results and Discussion

**Figure 2:**
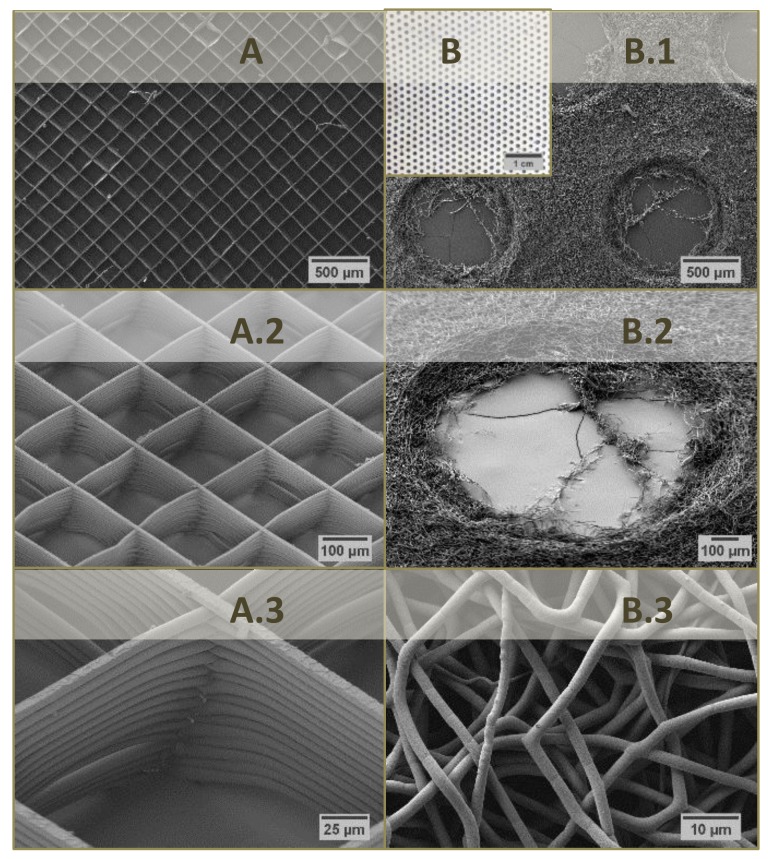
Scanning electron micrographs of MEW fiber mats from PCL with 30 fiber layers and a fiber distance of 200 (**A**–**A.3**) as well as scanning electron micrographs of the collector (**B**) and SES fiber mat from PCL with round holes (Ø 1 mm) in alternating rows and a hole distance of 2 mm (**B.1**–**B.3**).

During MEW, charged polymer melts are extruded through a nozzle onto a grounded, movable (cooled) platform [14] such that well defined coherent structures from straight fibers can be obtained when jet speed and collector velocity are well coordinated. This allows a reproducible layer build-up of 3-dimensional lattices for biomedical purposes [15]. The demand for a low melting point and a high thermal stability restricts the application of MEW technique to thermoplastic polymers, whereas PCL with a melting point of 63 °C [14] has been thoroughly analyzed in this context [15,16,17]. In the case of SES, a solution of the polymer in an organic solvent (mixture) [12] such as chloroform/methanol [18,19] or dimethyl formamide/tetrahydrofuran is used for PCL processing [12,20,21]. As with MEW, a high voltage is then applied to continuously eject the polymer solution onto the grounded collector. Due to fluid bending instabilities, the ejected material is twisted during deposition onto the collector leading to a disordered non-woven mesh of randomized nano-scaled fibers [22]. Macroporous SES fiber mats can be produced by either using porogens [23,24] or a patterned collector. The latter allows a disturbance of the electric field so that the deposited fiber mat mimics this pattern in order to control SES scaffold porosity [22,25]. For this study, four kinds of fiber mats were processed from PCL: MEW lattices with 30 fiber layers each with a fiber diameter of approx. 8 µm and a fiber distance of 200, 500 and 1000 µm, respectively, as well as a perforated SES mat (fiber Ø 800 nm–2 µm) with round holes (Ø 1 mm) in alternating rows and a hole distance of 2 mm (Figure 2).

The as-fabricated scaffolds were used to build up a laminate of alternating layers of premixed cement paste and either 3 PCL scaffolds prepared by SES and different surface treatments, three layers of MEW scaffolds with 300, 500 or 1000 µm fiber distance or three, five or seven layers of MEW scaffolds with 500 µm fiber distance. This preparation regime is different from previous works by other authors, in which electrospun PCL [26] or PLGA [27] scaffolds were cut into ~3 mm pieces prior adding them to a water mixed cement paste. Although a mechanical reinforcement effect was observed, none of these composites would enable free flexibility and shaping until application. In contrast, the presented laminates here can be cut into arbitrary shape (bending rod, defect-specific etc.) and are flexible until contact with an aqueous solution, e.g., water, PBS, SBF, physiological fluid or others.

From a purely subjective perspective, the following observations were made during the processing of MEW scaffolds: An increase in fiber space led to an improved cement paste penetration but also to an increase in PCL scaffold deformation through the mechanical stress applied with the spatula. At low fiber spaces of 200 µm, the paste was not able to penetrate very well into the scaffolds so that a partial separation of single layers within the laminate was observed. It is supposed that lamination might not work with higher (n > 7) scaffold numbers. Further, the fabrication of PCL scaffold with this geometry was very time-consuming. An observation similar to MEW lattices with small fiber distance was made using the porous SES scaffolds. Even though the pore size was only marginally smaller compared to the lattice with corresponding fiber distance of 1000 µm (0.8 vs. 1.0 mm^2^) those scaffolds exhibited an 8-fold higher areal density (Table 1) due to the large pore distance of 2 mm. Thus, at least 33% more cement paste was needed to cover the whole surface. Because of the higher increase in composite thickness, only three SES scaffolds were layered within one laminate. In this case, scaffold deformation by spatula derived shear stresses did not occur.

**Figure 3:**
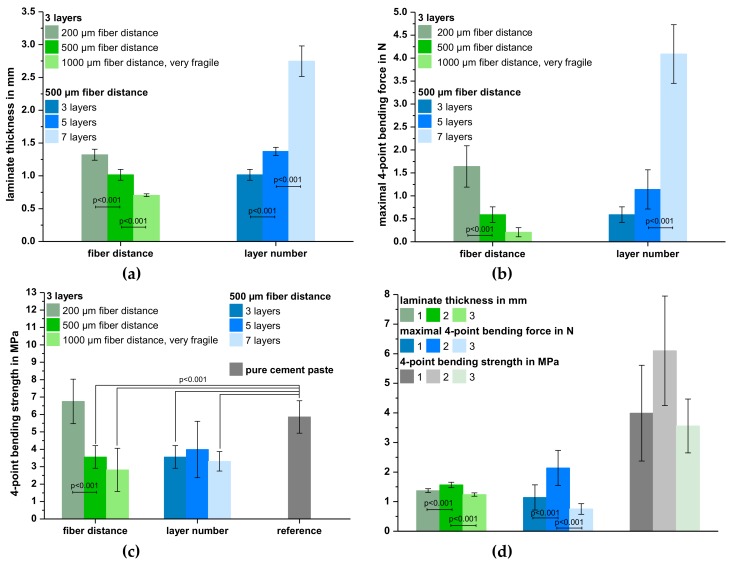
Laminate thickness (**a**), maximal 4-point bending force (**b**) and 4-point bending strength (**c**) of cured laminates from oil-based CPC paste and 3, 5 or 7 layers of MEW PCL scaffolds with a fiber distance of 200, 500 and 1000 µm. Reproducibility regarding the preparation regime was analyzed with respect to laminate thickness, maximal bending force and bending strength using laminates of oil-based CPC paste and five layers of MEW PCL scaffolds with a fiber distance of 500 µm as an example (**d**). The numbers describe the repetition of the experiment for preparing and analyzing laminates with the same composition and manufacturing regime.

The results of the characterization of the cured prefabricated laminates from cement paste and MEW scaffolds are depicted in Figure 3. The increase in fiber distance at a constant layer number of 3 resulted in a significant reduction of the laminate thickness from 1.3 ± 0.08 (200 µm fiber distance) to 0.71 ± 0.02 mm (1000 µm fiber distance). This is due to the fact that less cement paste was necessary to cover the whole construct. In contrast, increasing the layer number from three to seven layers at a constant fiber distance of 500 µm increased the laminate thickness by 170% from initially 1.0 ± 0.08 mm (Figure 3a). As expected, the maximum force of the 4-point bending test was proportional to the laminate thickness. A decrease in laminate thickness due to an increase of fiber distance reduced the maximum force by 87% from 1.6 ± 0.45 MPa (200 µm) to 0.21 ± 0.10 MPa (1000 µm). Simultaneously, an increase in laminate thickness led to a 7-fold increase in bending force with 4.1 ± 0.64 N for 7-layer composites (Figure 3b). However, calculation of the corresponding bending strengths revealed, that most formulations exhibited a comparable value of approximately 3.5 MPa, which was significantly lower compared to the pure cement paste (5.9 ± 0.93 MPa) after hardening under the same conditions (p < 0.001). Only using a small fiber distance of 200 µm and three layers or a bigger fiber distance of 500 µm with five layers resulted in laminates without significant difference compared to the reference group prepared from pure cement paste (Figure 3c). Although the prefabricated laminates with scaffolds of the smallest fiber distance showed the best mechanical results, it has to be mentioned that the fabrication of such small structures via MEW was very time-consuming. Thus, reproducibility of the preparation regime was tested by using laminates with five layers of the 500 µm-scaffold as they also exhibited satisfying mechanical properties. Even though the bending strengths were comparable in all three attempts, considering standard deviations, there were partially significant differences regarding laminate thickness and maximum bending force (Figure 3d). This shows that there is still a need for optimizing the preparation technique.

**Figure 4:**
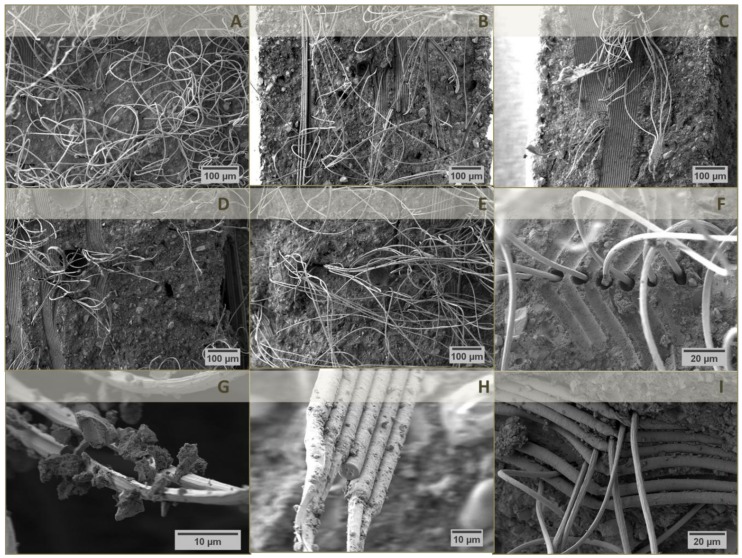
Scanning electron micrographs of fracture surfaces of cured laminates from oil-based CPC paste and three (**A**–**C**), five (**D**) or seven (**E**) layers of MEW PCL scaffolds with a fiber distance of 200 (**A**), 500 (**B**,**D**,**E**) and 1000 µm (**C**). Regions of interest were recorded at higher magnifications (**F**–**I**) for 200 µm fiber distance.

Fracture surfaces of the hardened composite specimens were analyzed by SEM (Figure 4). The micrographs revealed that the samples tended to break at the lattice junctions (Figure 4A–E) in a way that single fibers protruded either from the gaps between imprints of initially crossing fiber bundles (Figure 4F) or even from the gaps of crossing fiber bundles themselves (Figure 4G). Thus, the distance between top-down running (imprints of) fiber bundles might be used to estimate the thickness of single cement layers. As already mentioned, increasing the fiber distance of MEW PCL lattices resulted in a decrease in composite thickness (Figure 4A–C) due to removal of excess cement paste for a higher fiber distance, which in turn reduced the thickness of single cement layers between two PCL scaffolds. This amounted to 100–200 µm for a 200 µm fiber distance (Figure 4A), 70–150 µm for a 500 µm fiber distance (Figure 4B) while the layer thickness was not evaluable for a 1000 µm fiber distance as the fiber imprints of two PCL scaffolds nearly touched (Figure 4C). Obviously, the distance between two single PCL lattices varied widely which can be attributed to the mechanical shear stresses on the scaffolds during preparation. In addition, an increase in fiber distance simultaneously reduced the density of protruding fibers (Figure 4A–C). Having a closer look at the protruding fibers (Figure 4F–G), their diameter was approximately 50% smaller compared to the pores they left within the cement matrix (Figure 4F) as well as their original sizes. This observation is a consequence of the fiber stretching while bending of the rods. A further example of the tapering effect on pulled fibers is given in Figure 4I.

Lastly, both Figure 4H,I illustrate the deficient interface between fiber surface and cement matrix, as only few and small loosely connected cement fragments were found around PCL fibers. On the other hand, it could be shown that the cement paste penetrated the small gaps between single fibers of a whole bundle (Figure 4H) leading to partially equal bending strengths compared to the pure hardened cement (Figure 3c).

Due to the strong distortion of the MEW lattices during laminate preparation, the question arose if it is worthwhile to use such highly ordered structures. Thus, further experiments were carried out using SES PCL scaffolds, whereby a semi-order structure was implemented using a collector with defined perforations. In addition, a surface modification of the scaffolds by immersion in sodium hydroxide solutions was investigated in order to improve the bonding between the hydrophobic fibers and the cement paste. Among the different approaches to effectively modify the surfaces of PCL scaffolds [12], immersion in NaOH has been shown to improve wettability [28,29]. The treatment also leads to fiber meshes, which can be coated retroactively via mineral deposition [28,30] or with biologically relevant substances such as covalently bound gelatin to improve cell adhesion and proliferation [31]. In the present study, the SES PCL fiber mats were surface-modified by incubation for 10, 30 and 60 min in 1.0 or 2.0 M NaOH. Chemically, hydroxyl and carboxyl (–COOH) groups are introduced so that the surface is less hydrophobic while the fiber morphology is simultaneously modified [12].

**Figure 5:**
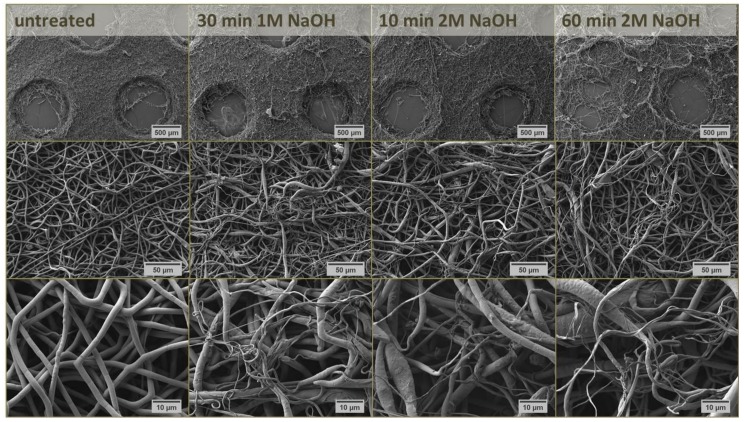
Scanning electron micrographs of SES scaffolds from PCL with round holes (Ø 1 mm) in alternating rows and a hole distance of 2 mm without treatment or after incubation for 10–60 min in 1.0 M or 2.0 M NaOH. Micrographs in the middle and bottom row are higher magnifications of the micrographs shown in the top row.

Macroscopically, increasing the NaOH concentration and duration of the treatment led to a strong disintegration of the semi-ordered structure (Figure 5). In this context, it seemed that the incubation with 1.0 M NaOH for 30 min had the same effect on scaffold morphology as the incubation with 2.0 M NaOH for 10 min (Figure 5). On a microscopic level, the homogeneous distribution of fiber diameters (800 nm–2 µm) of unmodified scaffolds (Figure 5) changed significantly by the treatment. Independent of solution concentration and incubation time, a lot of really thin fiber strands (~300 nm) were generated in addition to the swelling of fibers (~3.5 µm), leading to strongly irregular fiber diameters (Figure 5). Besides thinning of the fibers [32,33,34], other reports describe the occurrence of fiber morphology alterations [28], pits on the fiber surface [32,33,34] or even breakup of single filaments [29]. In spite of obvious structural differences compared to the untreated control, only marginal changes were observed with respect to the corresponding contact angles, especially for the lower concentrated NaOH solution (Table 2). Generally, water contact angles have to be <<90° for good wettability and ~0° for complete wetting of the surface [35]. This was only the case for a 60 min treatment with 2.0 M NaOH (Table 2). Thus, chemical treatment with an alkaline solution had a greater impact on the scaffold topography and fiber morphology than on the material wettability.

**Figure 6:**
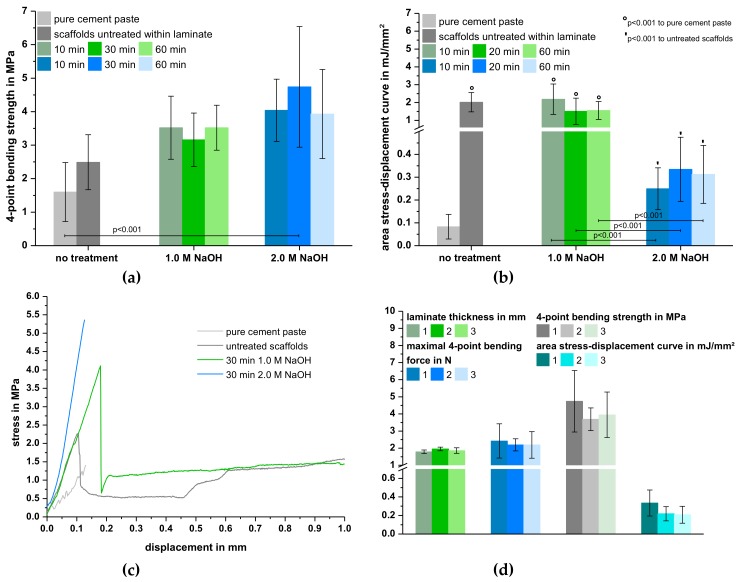
4-point bending strength (**a**), area under the stress-displacement curves (**b**) and selected stress-displacement curves (**c**) of cured laminates from oil-based CPC paste and 3 layers of SES PCL scaffolds with and without treatment in 1.0 or 2.0 M NaOH. Exemplarily, reproducibility regarding the preparation regime was analyzed with respect to laminate thickness, maximal bending force, bending strength and area under the force-displacement curves was analyzed using laminates of oil-based CPC paste and 3 layers of SES PCL scaffolds treated with 2.0 M NaOH for 30 min (**d**).

Results of the 4-point bending test of laminates with unmodified or NaOH-treated SES PCL fiber mats are shown in Figure 6. For the laminates investigated, the number of SES scaffolds was kept constant at 3 layers. Thus, no significant differences in laminate thickness (~1.7 mm) were observed, which was also comparable to prefabricated laminates with five layers of MEW scaffolds. This was because more cement paste was necessary to cover the whole surface due to the higher areal density of the SES fiber mats (Table 1). Furthermore, bending strength slightly increased with increasing NaOH concentration (Figure 6a). On the other hand, the immersion time had no effect on the mechanical stability. The laminates with untreated SES PCL scaffolds exhibited a bending strength of 2.5 ± 0.82 MPa whereas treatment with 1.0 M and 2.0 M NaOH resulted in values of approximately 3.5 MPa and 4.5 MPa, respectively. Hence, an increase in the hydrophilicity of the scaffold fibers, as shown by water contact angle measurements (Table 2), seemed to steadily improve the bending strength. However, significant differences compared to the cement control group could only be observed for laminates with PCL fiber mats incubated for 30 min in 2.0 M NaOH, which resulted in an almost 3-fold increase of the 4-point bending strength.

In contrast, evaluation of the area under the corresponding stress-displacement curves as a measure for the absorbed fracture energy showed different results. Only laminates with untreated scaffolds or scaffolds incubated in lower concentrated NaOH were able to absorb significantly more energy compared to the cement paste control. More specifically, the measured values between 1.5 ± 0.74 (30 min 1.0 M NaOH) and 2.2 ± 0.85 mJ/mm^2^ (10 min 1.0 M NaOH) corresponded to a reinforcement by one order of magnitude (Figure 6b). Furthermore, those formulations exhibited stress-displacement curves indicating additional force absorption after an initial rupture of the cement matrix. In contrast, the pure cement control as well as formulations with PCL scaffolds that were immersed in 2.0 M NaOH showed a ceramic-like, brittle fracture behavior (Figure 6c). This explains why the corresponding areas under the stress-displacement curves were—with values between 0.25 ± 0.09 (10 min 2.0 M NaOH) to 0.33 ± 0.14 mJ/mm^2^ (30 min 2.0 M NaOH)—about 80–90% lower. Overall, a variation of the incubation time in NaOH had only minor effects on the mechanical behavior (Figure 6b). Thus, reproducibility of the preparation regime with respect to laminate thickness, maximum force, bending strength and area under the force displacement-curves was investigated on laminates with scaffolds treated with 2.0M NaOH for 30 min, whereas no significant differences were found (Figure 6d).

**Figure 7:**
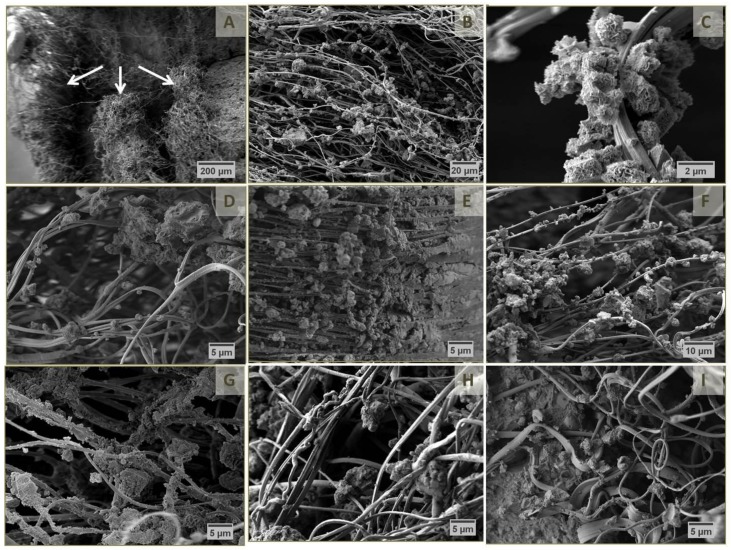
Scanning electron micrographs of fracture surfaces of cured laminates from oil-based CPC paste and three layers of untreated SES PCL scaffolds (**A**–**C**) or scaffolds after treatment for 30 min in 1 M NaOH (**D**,**E**), 10 min in 2 M NaOH (**F**,**G**), 30 min in 2 M NaOH (**H**) and 60 min in 2 M NaOH (**I**). White arrows in (**A**) indicate the three layers of PCL fiber mats in the composite.

To correlate the relation between interface and mechanical behavior, the laminates were also characterized by scanning electron microscopy (Figure 7).

A typical fracture surface of the cured laminates from SES PCL scaffolds and cement paste is given in Figure 7A–C, whereas the morphology was comparable among all laminates analyzed. The three layers of PCL fiber mats were notably distinguishable but appeared quite disordered due to the high number of nanosized filaments. Even without any surface treatment, noticeable accumulations of CaP crystals were found around single PCL filaments. In contrast to the CaP residues around MEW fibers (Figure 4F,I), these aggregates really seemed to have grown along the surface of the much smaller fibers. 30 min incubation in low concentrated alkaline solution further improved the interface bonding, e.g., intertwining of both components, even though no clear decrease in water contact angle was measurable (Table 2, Figure 7D,E). In case of the scaffold treatment in 2.0 M NaOH (Figure 7F–I), parts of the PCL construct seemed to be almost completely mineralized and surrounded by a cohesive CaP matrix in the laminates, which can be explained by the better wettability of the PCL fibers (Table 2). However, longer treatment in 2.0 M NaOH solution strongly weakened the interface, as the surface was too hydrophilic for a uniform bonding of fiber and oily cement paste (Table 2, Figure 7H–I). An effective fiber reinforcement can result in an improved strength as well as work of fracture [36]. For this, the interface has to be strong enough to enable appropriate load transfer and energy dissipation. The presented results have shown that SES PCL fiber mats already intrinsically provide a suitable interface (Figure 7B,C). Mild surface modification under alkaline condition was able to further improve bonding (Figure 7D,E) leading to reinforcement of the cement (Figure 6a–c). However, a too strong fiber-matrix interaction, which was observed for prefabricated laminates with scaffolds treated for 30 min in 2.0 M NaOH (Figure 7H), probably avoids potential energy dissipation in form of fiber pull-out or friction. In that way the resulting specimen had adequate strength, supposedly due to a reduced cement porosity, but likewise a conventional brittle fracture behavior, as seen in Figure 6a,c. Here, it was possible to increase the bending strength compared to the pure cement control group (Figure 6a), as the nanosized fibers might be able to densify the cement matrix which intrinsically contains an oil phase acting as leachable porogen [37].

In direct comparison of both approaches, scaffolds produced by MEW seemed to be less useful for the production of the presented laminates. The process only allows manufacturing of scaffolds with a low number of relatively thick fibers. Thus, the resulting contact area between PCL and cement is comparatively low in the laminates. In combination with the weak interface and the overall low fiber volume content, this leads to a deterioration of the mechanical properties of the cement. Additionally, scaffolds produced with this technique are easily distorted by shear stresses so that the benefit of geometric accuracy is compensated in this special application. In contrast, the high number of nanofibers within SES PCL scaffolds offers a large contact area for the cement matrix and in combination with the illustrated stronger interface interaction and overall higher fiber volume content a unique opportunity to merge a classical reinforcement strategy for CPC with the novel application form of a prefabricated, flexible and flat bone substitute.

## 4. Conclusions

The presented layer-by-layer technique was successfully used to produce flexible laminates from oil-based cement paste and PCL fiber mats with defined pore architecture. It was shown that SES with a patterned collector was more suitable for the fabrication of appropriate polymer scaffolds as compared to MEW, as fiber-matrix interface and thus mechanical performance was notably enhanced. Furthermore, PCL filaments initially prevent cement paste disintegration during processing and provide mechanical reinforcement within the hardened ceramic. During application, the setting time of the cement matrix of approx. 3–5 min (according to [10]) would result in a fast hardening of such prefabricated laminates after adaption to the wet bone defect. Since the set matrix is predominantly composed of HA [10], the implants will then only be degraded in vivo by osteoclast activity giving rise to prolonged resorption rates and mechanical stabilization of the defect site. Furthermore, the electrospun PCL fiber mats may degrade faster than the cement, gradually leading to bigger interconnecting channels, which can be infiltrated by cells and thus support bone bonding and incorporation into the surrounding tissue. Thus, the described laminates might be a promising alternative for the treatment of planar or curved cranial defects, where currently patient specific metallic or polymeric implants are used. In this context, the material approach described herein may simplify the treatment for smaller defects and will also avoid the implantation of non-degradable materials.

## Figures and Tables

**Table 1:** Areal density of scaffolds from PCL fabricated by MEW and SES with different porous structure. Except for the ♦ marked scaffolds, each scaffold exhibited significantly different areal densities with *p* < 0.001 to each other.

Scaffold Type	Areal Density [mg/cm^2^]
MEW, 200 µm fiber distance	0.91 ± 0.05
MEW, 500 µm fiber distance	0.54 ± 0.04 ♦
MEW, 1000 µm fiber distance	0.20 ± 0.03 ♦
SES	1.57 ± 0.11

**Table 2:** Contact angles of the surfaces of porous SES scaffolds from PCL without treatment or after incubation for 10, 30 or 60 min in 1.0 or 2.0 M NaOH. In case of ♦ marked scaffolds, no contact angle could be measured as the water droplet directly spread on the surfaces. The water drop was either deposited directly above a pore or on the scaffolds area between the pores.

Fiber Pretreatment		Contact Angle	
NaOH Concentration	Incubation Time	Above Pores	Between Pores
/	/	157 ± 7 °	156 ± 6 °
	10 min	128 ± 5 °	141 ± 5 °
1.0 M	20 min	136 ± 9 °	136 ± 5 °
	60 min	142 ± 2 °	145 ± 3 °
	10 min	♦	155 ± 6 °
2.0 M	20 min	♦	154 ± 10 °
	60 min	♦	♦
